# Collectanea

**Published:** 1831-08

**Authors:** 


### COLLECTANEA.

Floriferis ut apes in saltibus omnia libant,

Omnia nos, itidem, depascimur aurea dicta.

### PATHOLOGY.

*Case of Hydrothorax, in a Child fifteen months old.* By M. Lichtenstadt. (*Litt. Annal. de gesammten Heilkunde*, and *Bull. des Sc. Med.*)

Dropsical affections in the brain are, it is well known, most frequent during infancy; anasarca and ascites are occasionally seen after scarlatina; but we are not aware that any author has previously spoken of hydrothorax occurring during the early periods of life. The case related by M. L. is that of a well-formed child, fifteen months old, who, without any appreciable cause, was suddenly attacked with oppression of the chest and great anxiety: the heart pulsated strongly and irregularly; the little patient could not remain in a horizontal position, but remained in a sitting posture while the malady lasted, which was but a very short time, for it terminated fatally in a few hours. Upon dissection, both sides of the chest were completely filled with a limpid fluid; there was also a similar effusion within the pericardium. Neither the pleura nor the pericardium presented the least signs of inflammation. No particular appearances were observed in any other parts of the body.

---

*Cases of Spinal Irritation.* By Mr. David Wark, Surgeon, Dunlop. (*Glasgow Medical Journal.*)

Case I. 3d April, 1826. J. H. Weaver, ætat. forty-nine, of shattered constitution. Complains of dull pain at the breast, with incessant cough, almost preventing sleep, copious muco-purulent expectoration, dyspnœa, palpitation of heart, headach, and profuse nocturnal sweats; pulse ninety-five, bowels confined: has been ill four months, and treated with blisters to the breast, and cough-mixtures, without benefit. Dorsal vertebræ, about sixth, seventh, and eighth, are painful on pressure; pain stretching forward to breast, so acutely as to cause him to cry out. He had his bowels freely opened with purgative medicine, was confined to the horizontal position, and a blister was applied over pained part.

8th April. Blister, after several applications, discharges freely, but has produced a good deal of febrile excitement, which is subsiding.

12th. Blistered surfaces healed, and all the symptoms mitigated; pain in spine confined to one spot. The blister repeated, and kept open about eight days, restored him to his usual good health.

This patient had been given up for consumption, and certainly he bore some marks of phthisis: it appeared, however, to be merely chronic bronchitis, combined with spinal irritation.

Case II. 22d June, 1828. H. B. æt. twenty-one, a woman of stout habit, has been subject to cough for several years; for eight or nine months has complained of pain in the right side of chest, nearly constant, increased on inspiration, and coughing; weight and oppression at breast, difficulty of breathing, dry convulsive cough, occasional headach, and dullness of spirits. Pulse ninety, full, tongue moist, bowels natural, catamenia regular. Was bled to twelve ounces, and had a small blister applied to the breast, with very little relief.

3d July. Symptoms worse. The third, fourth, and fifth dorsal vertebræ are tender on pressure, particularly on right side; pain stretching acutely forward along the course of the intercostal nerves, to pained part inside the chest, causing dreadful convulsive coughing. The horizontal position was strictly enjoined, and twelve leeches ordered to pained part of spine, which gave immediate relief: this was followed by a small blister, kept open for a few days.

10th. Expresses herself greatly relieved: cough and other symptoms nearly gone. Three leeches more completed the cure.

About a year and a half afterwards, this woman being attacked with smallpox, the same symptoms recurred, but subsided with the fever.

Case III. 5th June, 1829. Mrs. A., æt. thirty-three, mother of six children, of delicate constitution, complains of intense pain on right side of head, which appears a little swollen; dry cough; pain and oppression at breast, little increased on deep inspiration; respiration hurried and laborious; pain and numbness about shoulders, stretching down arms; palpitation of heart; great debility, is fatigued on the slightest exertion, or even speaking; pulse 112, weak and irritable; bowels costive. Upper dorsal and lower cervical vertebræ are painful on pressure, most severe about third and fourth dorsal; pressure aggravating symptoms. Had a child about three months ago, and did not recover well; three weeks afterwards was affected with violent pain at breast, for which she was three times bled, and as often blistered, with but partial relief. Has consulted three medical men, who uniformly recommended blistering the breast. Is much reduced in body, and considered by herself and friends to be consumptive. Three days previous to seeing her, she had come fifty miles by land and water, for the benefit of sea air, and had caught cold on her passage, to which she attributes the aggravation of her complaints. Day before this had, of her own accord, applied twelve leeches to side of head, without benefit.

She had two blue pills at bedtime, followed in the morning by a full dose of salts and senna, which procured copious evacuations, with abatement of headach and febrile symptoms. Was ordered twelve leeches to upper part of dorsal vertebræ, to be followed by a blister.

As she lived a considerable distance from me, it was ten days before I again saw her. She was now so much better, that she was able to walk about without fatigue; appetite and strength improving every day: is still suckling her child. Says she has not enjoyed such health since delivery, and describes her feelings after leeching as if something were awanting about her breast. Blistered surface has discharged well, but is now healed; pain in spine confined to between third and fourth dorsal vertebræ, and much easier. Nine leeches, and a small blister, about the size of a crown-piece, kept open about eight days, removed her whole train of symptoms. I saw her about four months afterwards with slight return of same complaint, which was easily cured by the same treatment.

In this case the horizontal position was only enjoined a few days at first: in some cases the horizontal position is a *sine qua non* in the treatment, in others it is by no means essential.

Case IV. 28th August, 1829. I was called to Miss B., æt. twenty, of delicate habit, who said she was ashamed to see me, as she could not tell what she had to complain of, only she felt weak, and her appetite was gone. Pulse eighty, feeble, tongue moist, bowels natural, catamenia regular; stoops much, body reduced to a skeleton, so dull in spirits that she can scarcely be roused to the least exertion. On strict interrogation, admits having a slight feeling of weakness or weariness at breast. Upper dorsal vertebræ are tender on pressure, most about the fourth on left side; right quite free from pain; pressure aggravating symptoms at breast. About six weeks ago, after assisting the maids a short time at a washing, her hands and forearms became covered with a florid eruption, which soon disappeared, and was succeeded by a slight cough and uneasiness about chest, which have since gradually worn away. Was treated with a solution of Tart. Antim., bark, and other tonics, but without effect. Six leeches were ordered to pained part of spine, which procured immediate relief.

She was now sensible she had been labouring under more oppression at breast than she had been aware of. A small blister produced such constitutional derangement, and aggravated the symptoms so much, that I did not think of reapplying it. A few leeches were applied every second day for awhile, making in all thirty-one, which, along with the horizontal position greater part of the day, effected complete recovery. During the application of the leeches, she uniformly felt herself getting better; symptoms returning a little before next application, which gradually wore off towards the end. In less than four weeks her health and strength were completely restored.

Case V. M. C., æt. twenty-three. In summer, 1826, I attended this girl in fever: she was advanced in the disease, and had been neglected before I saw her; was treated with local bleeding, blistering, emetics, purgatives, &c., as symptoms indicated. Her recovery was slow, and accompanied with a host of nervous and hysterical symptoms, which have continued more or less ever since.

About two years ago, the abdomen getting enormously distended, and communicating a doughy feel to the fingers, there was little reason to doubt that her bowels were loaded with feculent matter. A course of purgative medicine was ordered, which brought away a prodigious quantity of dark pitchy-looking fæces, mixed with mucous and slimy matter: the belly, however, continued nearly of the same size, but a little softer; the stools were less in quantity, but much the same in appearance. Her strength getting exhausted, and her faith having failed her, I was obliged to abandon the practice. About six months after this she complained of pain in right hypochondriac region, aggravated on pressure, with frequent attacks of bilious vomiting: upon questioning her, she admitted having pain about shoulders, particularly on right side. Several medical gentlemen saw her, and she was more than once blistered over the region of the liver, and salivated with mercury, to no purpose.

On the 18th December, 1829, I was again called to see her. She had been getting worse for some months, and is now confined to bed. Complains of pain and giddiness of head, pain and numbness about shoulders and arms, particularly right arm; dull pain over region of liver and abdomen, most acute about caput coli, occasionally stretching down thighs: is much harassed with vomiting of acrid bile; eyes weak; speech has been hesitating for some months, is worse of late, stops often in the middle of words; abdomen reduced to natural size and feel; bowels open; pulse eighty, weak; menses have made their appearance all along, but a little irregularly. Says she is pretty easy while lying in the horizontal position, but all her symptoms are aggravated on getting up: gets so faint in the erect position, that she is soon obliged to lie down. These symptoms led me to expect the spine to be in fault: it was accordingly examined, and found tender throughout its whole extent, but particularly the cervical, lower dorsal, and middle of lumbar vertebræ; pressure, or the application of a sponge dipped in hot water, on the lumbar vertebræ, gave pain, aggravating pain in abdomen, and particularly at caput coli; pain shooting down thighs, along the course of crural nerves. On pressing the inferior dorsal, pain stretches forward to right hypochondriac region, which she describes as distinctly the pain she has so long felt there: pressure on the inferior cervical produced a feeling of pain and numbness about shoulders, stretching down right arm, which has not had proper feeling for some months: but the most remarkable symptom of all is in the upper cervical; slight pressure there increases the shooting pains over the head, and causes a feeling of constriction about the throat, increasing the impediment in speech, and causing difficulty of respiration. When the pressure is increased, the pain becomes intolerable, the function of voice ceases, and the respiration is as completely stopped as if she were suspended by a rope round the neck. Whatever part of the spine was pressed on, pain was felt shooting along the course of the nerves, but most severe on the right side. The upper cervical and inferior dorsal were the two points most severely affected, and from which I judged it not unlikely the pain might spread along the spine: these I resolved first to attack. Six leeches were applied to upper cervical, and same number to lower dorsal vertebræ. These were repeated with relief, and two small blisters were afterwards applied. In five days, when I again visited her, I found that the leeches had bled very freely, and had produced considerable debility: her face was pale and blanched, and she could with difficulty turn in bed. The blistered surfaces discharged for about a fortnight. It was a month before she gathered much strength: these symptoms, however, were mitigated, and she spoke more freely. By the beginning of April she was able to be out of bed the greater part of the day, spoke without hesitation, and was nearly free from former symptoms, but dorsal vertebræ, between ninth and tenth, were still a little tender. By the middle of June she could take exercise out of doors, had a good appetite; and the spine was sound, except between ninth and tenth dorsal vertebræ, where there still was tenderness on pressure, shooting through to right side, in which she still felt some uneasiness. Considered herself in better health than at any time since attack of fever.

The horizontal position may have been a good adjunct here, but that it was essential to the cure does not appear, as she was obliged to keep it most of the time for nearly six weeks before the treatment commenced, notwithstanding which she became every day worse. That the origin of the nervous system was in fault since fever, I doubt not, and that timely detection and timely treatment might have saved her from nearly four years' suffering and misery, and preserved her constitution from a shock from which it can never fairly rally, I as little doubt. The pain in side and shoulders and vomiting of acrid bile, were certainly symptoms of inflammation of the liver, but it is plain it was merely suffering in function, from disease of its nerves, as the heart and stomach are often known to do from the same cause.

Case VI. A few weeks ago I was called to see a young woman twenty-one years of age, whose prominent symptom was vomiting of every thing she took. She had pain in right hypochondriac region, increased on pressure, and pains about shoulders, shooting down right arm, which she describes as stitches. Had a child in the sixteenth year of her age, from which she dates the commencement of pain in side; pain in shoulders more recent; dyspepsia of some years' standing, but vomiting has only been distressing of late. Has been often bled and blistered for pain in side (supposed to be hepatitis,) and sometimes with partial relief. Had consulted a medical practitioner a few days ago, who ordered a large blister to be applied over region of liver. Ninth and tenth dorsal, and fourth, fifth, and sixth cervical vertebræ, are painful on pressure, the pain stretching to pained part in side and shoulders. Nine leeches were immediately applied to ninth and tenth dorsal vertebræ, and, in a few days, same number to inferior cervical. Eight days after this, she came a distance of about four miles, to shew me how much improved she was. Vomiting gone, pain in the shoulders and side much better, lies in bed most easily on right side, which she has not been able to do since she had the child; pained parts in spine still a little tender. Leeches ordered to be reapplied.

I saw her about a week ago, stout in body and looking well. Says that she enjoys excellent health, to which she has been a stranger for more than five years. The horizontal position was not observed in this case.

I have only met with one case of this kind which defied remedial measures; the prominent symptom was tickling cough: time, however, effected the cure. Several cases have been relieved, although they could not be said to be cured. This disease sometimes accompanies consumption, yet in one case I had strong reason to believe that it roused up fatal tubercular phthisis.

That this class of complaints is seldom seen, except in the debilitated walks of life, appears to be unfounded. Any thing here, in place of a town, scarcely deserves the name of a village. My practice is entirely in the country, in a place, too, famous for the salubrity of its air, and the healthiness of its inhabitants; yet in such a place spinal irritation holds no inconsiderable rank in the catalogue of human calamities.

As a stimulus to the younger candidate for medical eminence, I may be allowed to mention that, in the diagnosis and treatment of no other disease have I gained so much credit and confidence in families. I have cured several who have been long considered to be falling victims to consumption, the gaunt and unrelenting destroyer of mankind. Restored to the arms of their families and friends from a long period of hopeless sufferings, they often know not in what terms to express their gratitude.

---

*Fatality of Uterine Phlebitis. Great Importance of separating the Patients in the Wards of a Lying-in Hospital.*

Uterine phlebitis has been alarmingly frequent of late in the ward, St. Benjamin, in the Hôtel Dieu: it has generally come on violently, proceeded rapidly, and invariably been fatal in every instance, and under every variety of treatment. It is, however, now put beyond a doubt that the recent quick changes in the weather, with respect to temperature and humidity, have had much to do with it. With this impression, M. Caillard determined to take special care that his patients should enjoy a wholesome atmosphere; and, for this purpose, caused them to be regularly transferred to another apartment on the fourth day after delivery: thus keeping no more than eight or ten patients in the lying-in room, where there used, until now, to be about fourteen together at a time. Seventy-seven women have since been delivered, of whom but one has fallen a victim to phlebitis. Two others, on whom the forceps had to be employed, were, one of them attacked with metritis, the other with metro-peritonitis; but under the influence of antiphlogistic treatment, left the hospital as well and as healthy as the rest. The following table will shew the mortality, positive and comparative, of the several months since the beginning of the year. It should first, however, be mentioned, that the hygienic treatment above alluded to, was not adopted till the 22d of March.

*General Result, from January* 1, 1831, *to May* 23.

| | |
|---|---|
| Patients received into the ward St. Benjamin . . | 190 |
| Number of those who died . . . . . . . . . . | 17 |

January. Received, 45; died, 4.
February. Received, 38; died, 6.
March (to the 20th). Received, 30; died, 6.

*From March* 20*th to May* 23*d.*

Received, 77; died, 1.

Thus the mortality, which, in the first three months of the year, was one in about seven, became in the last two months, under the new system of treatment, on hygienic principles, one in seventy-seven.

With regard to the treatment most advisable in uterine phlebitis, it must be confessed that none in particular has been found efficacious, or deserving of preference. Bleeding, both local and general, though employed at an early stage, has been proved unsuccessful; and the antiphlogistic method has been found, in numerous cases, rather to hasten than retard the fatal result; though the bleeding, in fact, ought rather to have facilitated the transmission of purulent matter from the organ affected into the general circulation, and thus have materially served the patient. Nor did laxatives, mercurial frictions, or vesications, prove in the least more beneficial than the means just mentioned. The day may come, probably, when some specific may be discovered that may neutralize the deleterious effects of pus on the animal economy; but, in the present state of medical science, *hygiène* alone holds out any hope of succees for the prevention of this terrible disorder. These remarks apply also to the inflammation of veins which follows surgical operations, and which proves so often fatal. It should, however, be added, that M. Sanson, in cases of the latter sort, has experienced the best effects from the employment of tartar emetic; effects which place in a still more satisfactory point of view the excellence of that heroic remedy; though they require some little additional trial, in the crucible of experience, before they can be quite confidently relied on.—*Gazette des Hôpitaux*, and *Medical Gazette.*

M. SANSON'S *Practice. Phlebitis after Venesection: severe general Symptoms; Employment of Tartar Emetic in large doses; Cure.*

A PAVIOR, thirty-six years of age, a tall but feeble-looking man, was taken into the ward St. Jeane, at the Hôtel Dieu, on the 20th of April last, under M. SANSON. He had been affected with a violent pleurisy, under which he laboured for eighteen days, and for which he was bled in the left arm. The bleeding had the desired effect; but in three days inflammation of the arm came on, apparently in consequence of the puncture made by the lancet in the median-basilic vein. Leeches were twice applied to the part affected, and along the track of the vein; and after the leeches, cataplasms; but all in vain: the local symptoms became more serious, and on the fifth day the constitutional disturbance was so great that the man was obliged to be taken into the hospital. Besides the tumefaction of the subcutaneous cellular tissue, it was easy to feel along the course of the vein several little collections of matter. The most excruciating pain, too, was felt by the patient in the armpit of the affected side. The limb had acquired a stiffness, and an extreme sensibility; and among the general symptoms were horripilation, fever, headach, inexpressible uneasiness, and a troublesome cough, which seemed to proceed from disorder of the thoracic viscera. Five and twenty leeches, after which emollient fomentations to the whole of the arm. Laxative drinks. The next day, and the next, the same remedies were employed; but the general symptoms, so far from abating, became so serious by the fourth day, that M. Sanson thought himself bound to have recourse to that remedy which he has so often used with so much advantage; he ordered Tartar Emetic, eight grains to the dose, given in four ounces of julep, *(infusion de tilleul edulcorée.)* The patient took the whole of the mixture in the course of the day without experiencing any nausea; and the next day it was repeated. From that time every threatening symptom disappeared, and along with the constitutional affections, the local ones: the collections of purulent matter resolved rapidly, and the patient had no longer any pretext for confinement to his bed, except some little annoyance from the respiratory organs. On the 26th of May he left the hospital quite convalescent.—*Ibid.*

---

### PRACTICAL MEDICINE.

*Chlorate of Lime in Gonorrhœa.* Notwithstanding the long list of remedies we can boast of, for the treatment of gonorrhœa in all its forms and stages, it must be confessed that cases not unfrequently occur which perplex both the patient and practitioner, from the long endurance of troublesome and even painful symptoms. We must not, therefore, refuse to take advantage of any additional remedy, from the use of which we are assured much success has been obtained. In a recent number of the Lancet, it is said that the chloride of lime has been successfully administered as a remedy for gonorrhœa, and Mr. Clough states (Lancet, July 9th,) that he has been in the habit of employing it for some time past, not internally, but in the form of injection, with the most decidedly good effects. With the exception of a few doses of aperient medicine, he has latterly had recourse to no other remedy. The formula employed was two drachms of the chloride of lime to six ounces of distilled water. The practical value of Mr. Clough's communication would be increased, if he were to state whether the treatment he recommends is applicable to every form of the disease; and if it be not, he should inform us what particular symptoms it is calculated to relieve.

---

*Evidence in proof of a Remedy for the immediate Relief of Toothach when arising from Caries or decayed Teeth, without any Pain or the Operation of Extraction.* By Dr. Ryan.

Of the numerous diseases incidental to humanity, few are of more painful, or of more frequent occurrence, than toothach; and for none is there less sympathy, or less relief by medicine. The concurrent testimony of the scientific portion of the profession attests the truth of this position. A remedy which is capable of affording immediate relief to the excruciating pain of toothach, without the slightest pain being produced by its application, has long been a desideratum; and I feel great gratification in being the medium of proposing such a valuable remedy to the profession.

It is right to observe, that before I resolved upon this course, I deemed it necessary to determine the value of this agent, and to try it upon myself and many other individuals; and ample experience has convinced me of its efficacy.

Like many of our best remedies, that which I proceed to notice was discovered by accident. A gentleman who attends my lectures (Mr. Myers, of Newington Causeway), had frequently applied sulphuric acid to his tooth, with some relief; but on one occasion he, in a moment of confusion, took down the next bottle to his remedy, which contained nitric acid. To his great surprise, he experienced immediate relief, and without the slightest pain. Since that period he has not suffered from toothach, though three years have now elapsed. During the last winter he informed me of the success of this remedy, which induced me to try it, while labouring under the most intense pain from toothach. The effect was immediate, and no pain whatever was induced. I have since used it in numerous cases, and invariably with complete success. In some instances the disease does not return for days or weeks; and in others not for months.

The best mode of employing it is by means of lint wrapped round a probe, and moistened with the acid, which is then to be slowly applied to the cavity of the tooth; care being taken not to touch the other teeth, the gums, or the cheeks. On withdrawing the probe, and inquiring how the patient feels, the usual reply is, "the pain is entirely gone." The mouth is next to be washed with tepid water. The acid should be gradually applied to the whole cavity of the tooth, or otherwise a second application will be required before complete relief will be obtained.

This remedy may be used when the gum and cheek are inflamed, so as to preclude the possibility of extraction. In cases where the diseased fang remains, and when the caries faces the adjacent tooth, it obviates the necessity of extraction in all cases of hollow teeth, which all practitioners declare to be desirable, if possible; and it enables the dentist to perform the operation of "stopping or filling teeth," much sooner than he can otherwise accomplish. In a word, it will alleviate a vast deal of human suffering, and supersede a most painful operation. It is not a panacea for all the diseases of the teeth and gums, though a certain and efficacious remedy for the most common cause of toothach. It will be a valuable remedy for children, delicate persons, and pregnant women. It does not accelerate the decay of the tooth to which it is applied.

As the employment of this acid in the disease under notice is not recommended in any pharmacopœia, ancient or modern, of these or other countries with which I am acquainted, and as toothach is now a most prevalent complaint, in consequence of the inclemency of the season, I think a more favorable opportunity cannot occur for the communication of the information described in this paper. —*London Med. and Surg. Journal.*

---

*Colchicum in Rheumatism.* By ALEXANDER TWEEDIE, lately Clerk to the Clinical Physicians.

THOUGH colchicum has long maintained a very respectable rank in the list of our remedial agents for the cure of rheumatism, yet from time to time there have been some who have doubted its efficacy; and even when used with the happiest effects, the result has not been so speedy as to satisfy the sanguine expectations of many of its supporters. Much of this discrepancy of opinion is doubtless ascribable to the notorious fact, that the fluid preparations of the drug are compounded in many different ways by various practitioners, a circumstance of itself sufficient to produce great uncertainty of effect, and in many cases complete disappointment.

Another great cause of failure and uncertainty may perhaps consist in this, that the remedial principle of the drug is probably not entirely taken up by the menstrua in either of our pharmacopœial preparations; so that when the vinum, or the acetum colchici, disappoints our expectations, we are scarcely justified in condemning the drug as useless, whose specific principle has, under such circumstances, never been administered at all, or very partially. To obviate these objections it becomes necessary to administer the remedy *in substance*; and it is to shew the benefit of this plan of exhibition that I have ventured to trouble you with these remarks.

The form of application most commonly, I believe, adopted, and which has for some time past been practised by Dr. Bright in this hospital, is the same as that recommended in a recent number of your Gazette by Dr. Lowder: I mean the vinum colchici, combined with so much magnesia and Epsom salt as might suffice to procure several stools daily. Now this, though hitherto the most successful practice, was yet found to fail so frequently, to seem, in fact, so often inert, that Dr. Addison, our then clinical physician, was happy to avail himself of the evidence of Dr. Jackson, of Boston, in the United States, who was attending our clinical wards as a pupil, and who stated that in the hospital at Boston it was customary to administer the colchicum (for the cure of rheumatism) in substance, to the extent of half a drachm in twenty-four hours, combined with a little magnesia and salts, with such uniform good effect, that he had never had the opportunity of seeing any other plan of treatment called for or adopted, nor was depletion ever premised. Dr. Addison accordingly determined to give the remedy a fair trial, and the following cases, briefly related, will shew with what effect.

Case I. W. Gibson, a delicate fair boy, æt. ten, admitted 2d March, 1831. At the time of admission, the two wrists were the only parts affected with acute rheumatism, arising from exposure to cold a fortnight previously. His bowels were confined; pulse 100, full; tongue white, and great heat of skin. The vinum colchici was given, and an occasional dose of salts and senna with the vinum colchici, to keep the bowels free; but instead of decreasing, the disease gradually and steadily increased; other joints became involved; and notwithstanding the trial of quinine, opiates, antimonials, mercurials and salines, &c. one after the other, the disease continued to shift from one joint to another, and in the end to place him in a situation of considerable distress and suffering. On the 15th March the disease was at its height; both wrists and both ankles painfully inflamed, with less degree of pain in the loins and joints of the extremities generally; he could not move himself in bed without pain. Skin dry and hot; tongue coated, inclined to brown, and somewhat dry, red at the edges; bowels not open since yesterday; pulse 128, small and sharp. Considerable pain across the forehead; great thirst; no appetite.

The bowels were opened by means of an injection, and on the following day, March 16, he was in much the same state as on the day before, when the following was ordered: Pulv. Rad. Colchici gr. iv. sextis horis, superbibendo Haust. sequent. ℞. Mag. Subcarb. gr. x.; Magnes. Sulph. ℈ji.; Aquæ Menth. ℥j. M. fiat haust. ℞. Calomel gr. j.; Antim. Tart. gr. $\frac{1}{4}$; Opii Pulv. gr. $\frac{1}{2}$. M. fiat pil. h. s. s.

N.B. The above pill had been given for some nights previous, without benefit.

March 17th. Has rested perfectly well; one stool. The only remaining, and that a very trifling pain, affects the left shoulder; no headach; abdomen soft, slightly tender, with occasional griping pain at the lower belly; tongue furred, clean at the edges; thirst continues; complains of nausea since yesterday; pulse ninety, more expanded, softer; slight perspiration about the face and neck. Pergat.

18th. Slept well; a very slight pain remains in the shoulder only; he complains of pinchings in the hypogastric region, with some nausea. Two semifluid yellow dejections; tongue cleaner; skin almost cool; pulse down to sixty-six, softer, more expanded, and with a peculiar hesitation in its beat, not amounting to actual intermission; (the heart was similarly beating, slowly and hesitatingly.) Pupils dilated; aspect free from suffering, its expression quite composed; head and face perspiring.

Being thus completely under the influence of the colchicum, and the rheumatism having been entirely subdued, it was thought expedient to withhold it, and the patient was directed to be kept perfectly still in bed. Rep. Pil. h. s.

19th. Slept well; no dejection this morning; complains of slight nausea, with a little tenderness on pressure of epigastrium. Tongue scarcely furred; pulse sixty-six, less hesitating; appetite better. Has some griping pain in the lower belly, particularly during making water, with slight local tenderness on pressure. Quiescat.

21st. Rest good; no rheumatic pain; tongue and skin natural; bowels well open; pulse sixty-eight, regular and firmer; appetite better.

His convalescence was now established; he had no return of rheumatism; and as the effect of the colchicum subsided, his pulse acquired its natural character; he lost all symptom of illness, and was sufficiently strong to be discharged on the 29th March.

Case II. Susan Simson, æt. twenty-two, a married woman, of robust constitution, admitted 6th of April, 1831, with acute rheumatism of the right knee, and of the right wrist, extending into some of the joints of the adjacent fingers. It commenced a week ago, and has been gradually getting worse till now. Pulse 120, jerking and sharp; tongue thickly loaded with moist white fur; skin warm, not perspiring; bowels not open; very little headach.

n.b. She has been suckling her child up to the day of admission. Ordered, Pulv. Colchici gr. iv. quartis horis superbibendo haust. Magnesiæ c. Magnes. Sulphate.

7th. Five doses of the colchicum have been taken with the effect of procuring two not unhealthy stools without griping or nausea. The rheumatism in the hand and knee is less acute, but she has a fresh, very slight, seizure in the left knee this morning. Pulse ninety-two, softer, more expanded; tongue less loaded; surface temperate; no headach. Pergat.

8th. Rheumatism nearly quite gone; a very little tenderness of the right wrist and knee only remaining. Tongue more clean; pulse seventy-four, much softer; bowels open thrice; copious loose yellow stools. Pergat.

10th. Rheumatism gone; pulse sixty, expanded, and hesitating; four stools, of the same character as before; no headach. Hab. Colchicum sextis horis tantum.

From this time she had no relapse. She was well enough to sit up on the next day, and went out of the house quite well on 19th April.

Case III. R. Orton, admitted 30th March, a stout, dark-complexioned lad, the subject of rheumatism for three weeks. At the time of admission his wrists were swelled, stiff, and slightly red; and there was a less degree of inflammation in the shoulders. Pulse seventy-two, full and sharp; tongue thickly furred; no headach; not much thirst, or heat of skin; bowels open, but abdomen rather full. Pulv. Colchici, gr. iv. sextis horis superbibendo haust. Magnes. c. Magnes. Sulph.

March 31st. Slept well. Slight pain on motion only in the right shoulder and fingers. Pulse sixty-eight, less sharp, and wider. Three or four copious, thin, yellow stools.

April 1st. No pain. Some stiffness of the joints remains. Pulse fifty-eight, soft, and hesitating. Three dejections. Feels somewhat mentally depressed, with a little giddy sensation across the brows.

April 2d. Stiffness going off. Pulse fifty-two, of the same character. Two stools. Omit. medicamenta.

3d. Slept well, but there has come on a pretty smart relapse of rheumatism in the right wrist. Rep. Colch. et Mist. ut antea.

4th. A good night; the inflammation and pain of the wrist subsiding. Pulse sixty-four; one stool; tongue nearly clean.

6th. Rheumatism gone. Pulse fifty-four, expanded and hesitating.

He left off the medicine on the following day, and left the house well on the 12th.

Case IV. James Williams, æt. eighteen, a dark clear complexioned lad, admitted 23d March, with not severe rheumatism of the right shoulder and knee; the other joints being in still less degree affected.

The remarkable feature in this lad's case was an affection of the heart, the result of previous rheumatism, of which he had had several attacks. Five years ago he was laid up for two months with acute rheumatism in most of his joints. A year and and a half ago, he had another attack, that lasted eighteen weeks. He got up and exposed himself prematurely in his convalescence from this, and in consequence met with a relapse, with pain about the region of the heart (for the first time), frequent palpitations, and dyspnœa. He seems to have pretty well recovered from this, so as to be able to resume his occupation, which is quite sedentary, feeling no further inconvenience from his palpitation, &c. till his present illness, which came on five weeks ago. The rheumatic inflammation since then has been seizing, in irregular succession, nearly all the joints of his extremities, and was at its height a week ago; at which time only did he begin to experience any inconvenience from the palpitations of his heart, which have since then been at times very severe indeed.

At present, pulse 120, full and quick, not hard. The heart's beat is remarkably forcible, even to the eye, but it does not seem diffused beyond the immediate vicinity of the organ; a very slight bellows-sound attends its second stroke; respiration twenty-eight in the minute. He has slight cough; bowels natural; tongue not much furred; very little headach. Pulv. Colchici, gr. iv. sextis horis superbibendo Haust. Magnes. c. Mag. Sulph.

March 24th. Rested moderately well. The only remaining pain is trifling, and in the left shoulder. Pulse 104, more natural; respirations twenty-two, free; heart's impulse decidedly less forcible and diffused; no alvine evacuation. Pergat.

25th. No alteration. Pulse 100, softer; bowels not open. A dose or two of colchicum has been accidentally omitted. Haust. Sennæ st. et Pergat.

26th. Bowels well opened. Rheumatic pain all gone. The heart's beat more limited, and the bellows-sound fainter. Pulse ninety-two, softer. Pergat.

Towards evening the pulse sunk still lower, to sixty; and, as he was labouring under some affection of the heart, it was not considered prudent to push the colchicum further: it was accordingly discontinued. On the following day the patient was sitting up, and felt altogether so well, (the palpitations of the heart disturbing him but little, and his appetite far exceeding the bounds of hospital diet,) that he would remain in the house no longer, but left us of his own accord on the 30th.

*Remarks, &c.* The sum and substance of what has been already said by various writers respecting the treatment of rheumatism, has, I believe, pretty well established the fact that colchicum is its best remedy; and I think the above cases (only a portion of those similarly treated by Dr. Addison in our clinical ward) afford proofs of the powers of the drug, such as are seldom witnessed from the exhibition of any of its artificial preparations. When administered as above described, in doses proportioned to the age and strength of the patient, it for the most part exercises an influence on the system at once marked, decided, and beneficial. After four or five doses have been taken, this influence begins to shew itself: the pulse, which was before hard and frequent, diminishes in number very remarkably; it becomes at the same time softer and more expanded, and there is a hesitation in its beat truly characteristic. The pulse, in fact, resembles much that of oppression of the brain in its slowness and hesitation, but has none of its hardness. With this there is a sense of vertigo and dizziness, especially when the patient attempts to sit up. The pupils are sometimes dilated; there is occasionally, but not always, nausea; and the patient complains of uneasy griping sensations in the abdomen, which are chiefly referred to the hypogastric region, and always aggravated during the act of either of the evacuations. The stools are for the most part characteristic: they are of a peculiar loose, yellow nature, such as are seldom, I believe, seen under other circumstances.

In proportion as these symptoms indicate the influence of the colchicum on the system, the rheumatic pain becomes diminished, the swelling and inflammation subside, and in very brief space of time indeed (as evinced by the above-related cases,) the disease is at an end. The remedy being now discontinued, all its symptoms gradually decline, leaving the patient quite convalescent, and with a good appetite. But this remedy is by no means infallible.

When administered so as to make the impression on the system above detailed, and when the number of stools procured by its combination with the magnesia and salts does not exceed three or four daily, a rapid improvement takes place; but there are individuals who cannot be placed under this favorable influence. The exceptions to which I allude consist of those cases where the mucous membrane of the bowels is so irritable that sufficient doses of colchicum cannot be administered without inducing excessive purging; and it has been found in our clinical ward, that when such purging takes place, the system manifests none of the symptoms above mentioned, as indicative of the influence of the medicine. In such cases it would appear as if it expended its whole powers on the bowels, and became removed from the body by the operation of purging, before it had time to affect the general nervous and vascular systems in the peculiar manner on which seems to depend its favorable operation.

These exceptions, however, constitute only a minority; and I know of no rule by which they may, *à priori*, be known. Dr. Addison gave the colchicum in nearly every case of acute rheumatism indiscriminately: if purging supervened, he found no difficulty in arresting it by means of Dover's powder, &c. and in continuing the treatment accordingly by other remedies.

In very chronic, and in mercurial and syphilitic rheumatism, the colchicum, as here recommended, has not been successful, hypercatharsis being in some instances induced, and in others the remedy failing, even when symptoms have clearly indicated its impression on the system; but in the more acute cases we have had the opportunity of treating, except where the bowels have been too irritable to bear the remedy, we have not had a single instance of failure.

I would take this occasion to state, that after finding so successful a result to follow the exhibition of colchicum in this way in acute rheumatism, Dr. Addison gave it in several of those obstinate neuralgic abdominal pains, that constitute so great a proportion of female suffering; and though in general the individuals who are the subjects of such complaints were too irritable to bear it, yet we have had two or three cases where it seemed to have undoubted good effect. Dr. A. has not tried it in a sufficient number of cases whereon to found any decided opinion: he is, however, inclined to think favorably of it, and purposes giving it a fair trial, the result of which may possibly at some future period be communicated to you.—*Med. Gazette.*

MISCELLANEOUS.

*Poisoned Confectionary.* Dr. O'Shaughnessy has recently published in the Lancet, No. 402, a very interesting memoir upon this subject. Although it does not appear that the trash known by the name of "sugar confectionary" contains, in general, such a proportion of poisonous ingredients as to render it immediately dangerous, Dr. O'S. proves more than enough to shew the impropriety of allowing children to indulge in eating these "*bonbons*," as they are most inappropriately denominated.

Mr. Chevallier has recorded, in the Journal de Chimie, various serious accidents produced by the consumption of sugar confectionary coloured by mineral poisons. Of these he particularizes the *schweinfurt green*, a compound of arsenious acid (arsenic) and copper, the *chromate of lead*, and the *sulphuret of mercury;* lastly, he enumerates *gamboge*. As the practice continued, notwithstanding it was fully exposed in various French journals, the Council of Health was consulted on the subject, and ultimately an ordinance was issued for the suppression of the nuisance, in consequence of a report which M. Andral addressed to the prefect of police upon the subject.

Dr. O'Shaughnessy shews that this mischievous practice is not confined to France; for he purchased, at several shops in this metropolis, different specimens of coloured confectionary, and of colourless articles, wrapped in stained paper. Some of these articles were sold expressly for eating, and some were *apparently* intended for ornament, but were sold without restriction. Of ten specimens of red comfits, sold for eating expressly, six contained mineral poison: all these specimens, with one exception, were only coloured externally; the articles which were differently coloured, were also found to contain deleterious substances. Dr. O'S. had not leisure to determine the precise quantity of poisonous ingredients contained in the various articles he examined, but he very properly observes, that "the minutest possible quantity of any such substance ought not to be allowed." It appears, indeed, that the quantity of deleterious matter contained in coloured confectionary is sometimes sufficient to endanger life; "for, not long since, an *acute* case of poisoning, arising from the use of confectionary of this description, occurred in the children of a highly respectable family in Southwark, and, on analysis, the comfits were found to contain minium, or the red oxide of lead."

For the particular mode of analysing suspected specimens of confectionary, we must refer to Dr. O'Shaughnessy's paper. The zeal and intelligence with which this gentleman has investigated the subject, entitles him to the gratitude of the public and the esteem of the profession. Upon one point he is, we believe, in error: he laments that the *statute* law of England affords the public little protection against any system of the kind he deprecates, "no matter how deadly its nature." This may be true; but it must not be forgotten that the *common* law of the land is amply sufficient to punish offences of the sort as a public nuisance, although no express *statute* may exist in which they are formally included.

